# Internationalization

**DOI:** 10.1590/2176-9451.19.4.013-014.edt

**Published:** 2014

"To have a new language is to have a new soul." Juan Ramón Jiménez, Spanish poet.

Globalization and internationalization are terms of the corporate world. In this scenario, mastering the English language is indispensable and with Brazilian science is not different. CAPES (Coordination for the Improvement of Higher Education Personnel) and CNPq (National Council for Scientific and Technological Development) have encouraged postgraduate programs and scientific research to effusively enter into the international scenario. However, the Shakespearean language is the foundation of this building. CAPES and CNPq programs have advanced and are good examples, despite Brazilian one-legged basic education system. Science without Borders is a nationwide scholarship program funded by the Brazilian federal government and is a good example we should be proud of. The program has expanded and, nowadays, includes not only postgraduate, but also undergraduate programs. Nevertheless, supply exceeds demand, especially because most undergraduate and postgraduate students do not master the English language. The solution was to establish the program English without Borders.

With a worldwide, clinically and scientifically, renowned Dentistry, Brazilian journals have increasingly sought internationalization in the counterflow of federal support. Internationalization is, in general terms, the need for communicating in a single language.^1^ In Brazilian Dentistry, six out of the seven journals indexed in SCOPUS are fully published in English. That is how our Dental Press Journal of Orthodontics (DPJO), with an ear to the world, has been disclosed: in English, since 2010, as well as in Portuguese. This is a milestone in the internationalization of this journal which has been sculptured for nearly a decade.

Speaking does not guarantee us to be heard. For this reason, in addition to the need of establishing communication in the language shared by science, it is of paramount importance that the journal content be disclosed. Thus, step-by-step, DPJO was being embraced by the major international databases: SciELO (2005), SCOPUS (2008) and, recently, PubMed (2013). We acknowledge that publishing a journal in a universal language globally increases reader's interest. However, what would be the impact of this process of internationalization on overseas authors?

We are aware that the number of articles written by Brazilian orthodontists published in international journals has increased in the last decades.^2^ Nevertheless, the interest in publishing in Brazilian journals did not follow the same pace. In 2012, when DPJO was not yet indexed in PubMed, only 3% of articles were submitted by foreign researchers. These articles came from India, Malaysia and Iran and did not fulfill acceptable methodological quality. After being indexed in PubMed, the number of submissions by foreign authors grew dramatically. By analyzing data from the last twelve months (Aug, 2013 - Jul, 2014), we notice that one third of articles submitted to DPJO come from different countries such as Canada, the United States, Italy, Colombia, Pakistan, India, Mexico, Saudi Arabia, Singapore and Iran. An increase that surpasses 1000%. Within this period, DPJO acceptance rate was of 30% for articles submitted by Brazilian authors and 10% for overseas authors. These numbers lead us to infer that not only the amount of articles submitted by overseas researchers has increased, but also the number of high-quality articles reaching our brooks.

A new language renews the soul and provides those who seek leadership in a high-quality corporate world with courage. Therefore, our young journal has followed the steps of the Spanish poet cited in the epigraph whose exile led him to learn a new communication tool capable of spreading his art throughout the literary world. We go on publishing in the Portuguese language. Thus, we have not renounced the language of Camões, but acquired a new soul instead.
